# Supplementary figures and images for: Exploring the antimicrobial and antibiofilm potency of four essential oils against selected human pathogens using in vitro and in silico approaches
Source: PLoS One. 2025 Apr 24;20(4):e0315663. doi: 10.1371/journal.pone.0315663 (PMC12083874; doi:10.1371/journal.pone.0315663)

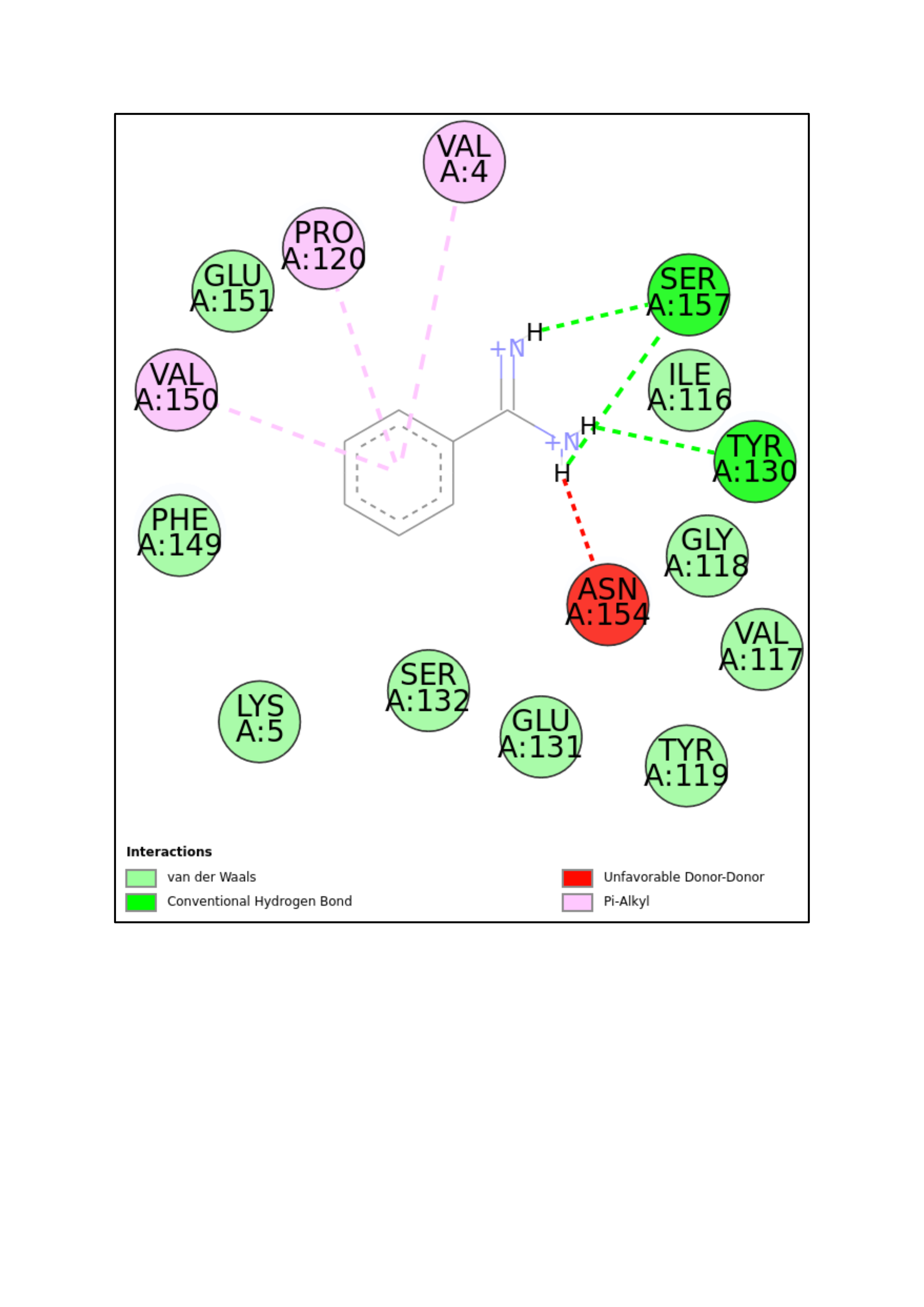

Supplement: S1 Fig — (TIF) [file pone.0315663.s001.tif]

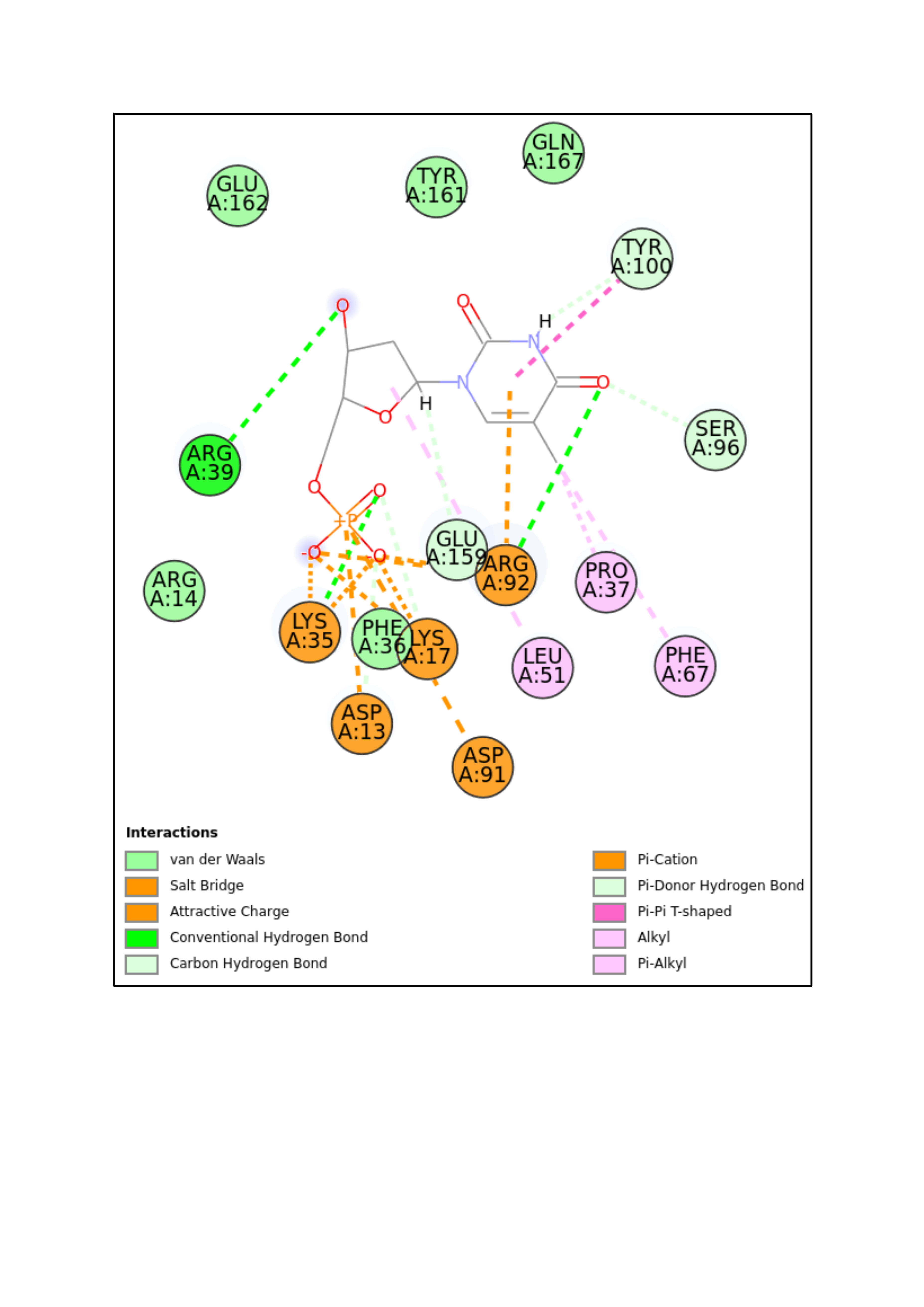

Supplement: S2 Fig — (TIF) [file pone.0315663.s002.tif]

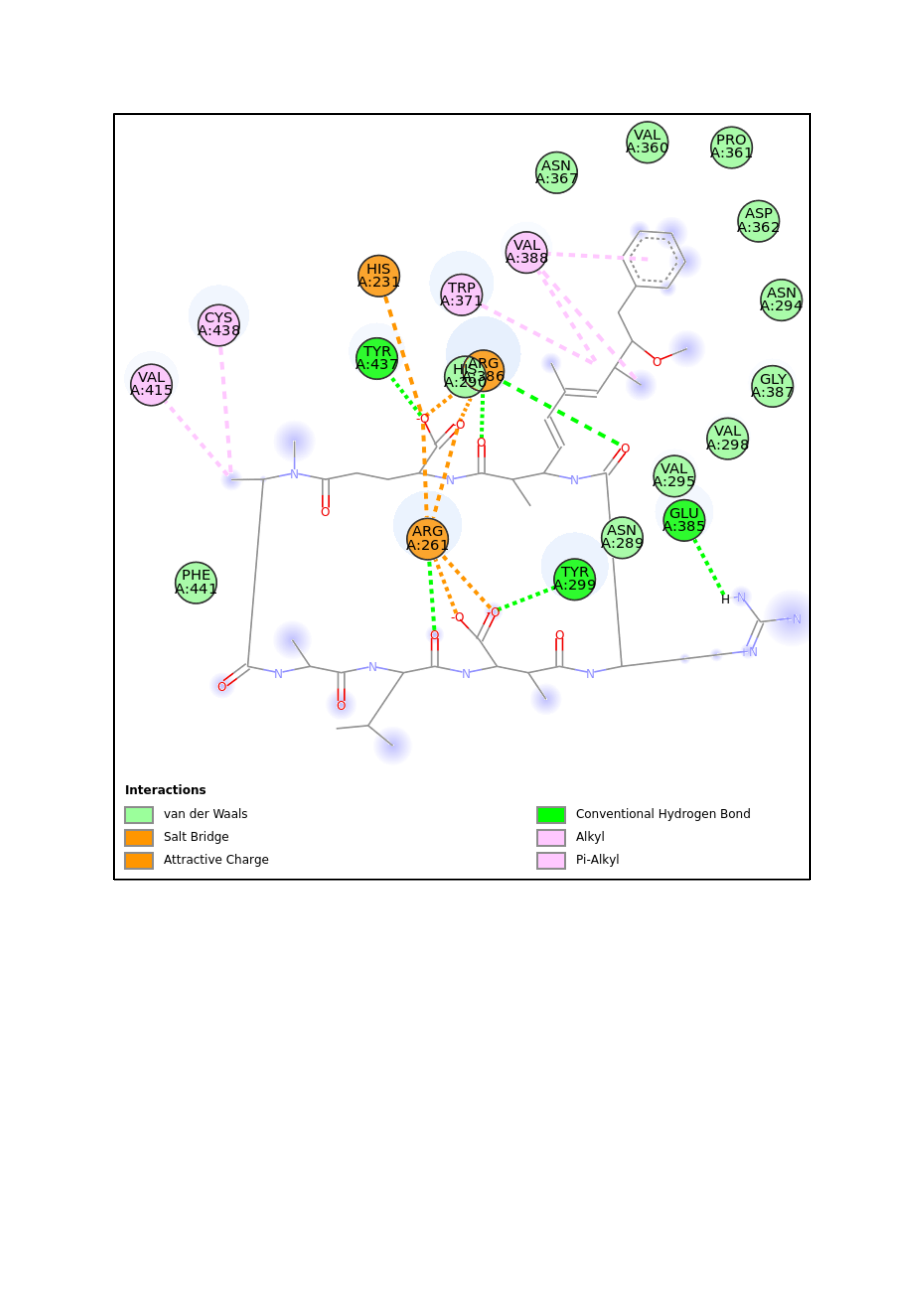

Supplement: S3 Fig — (TIF) [file pone.0315663.s003.tif]

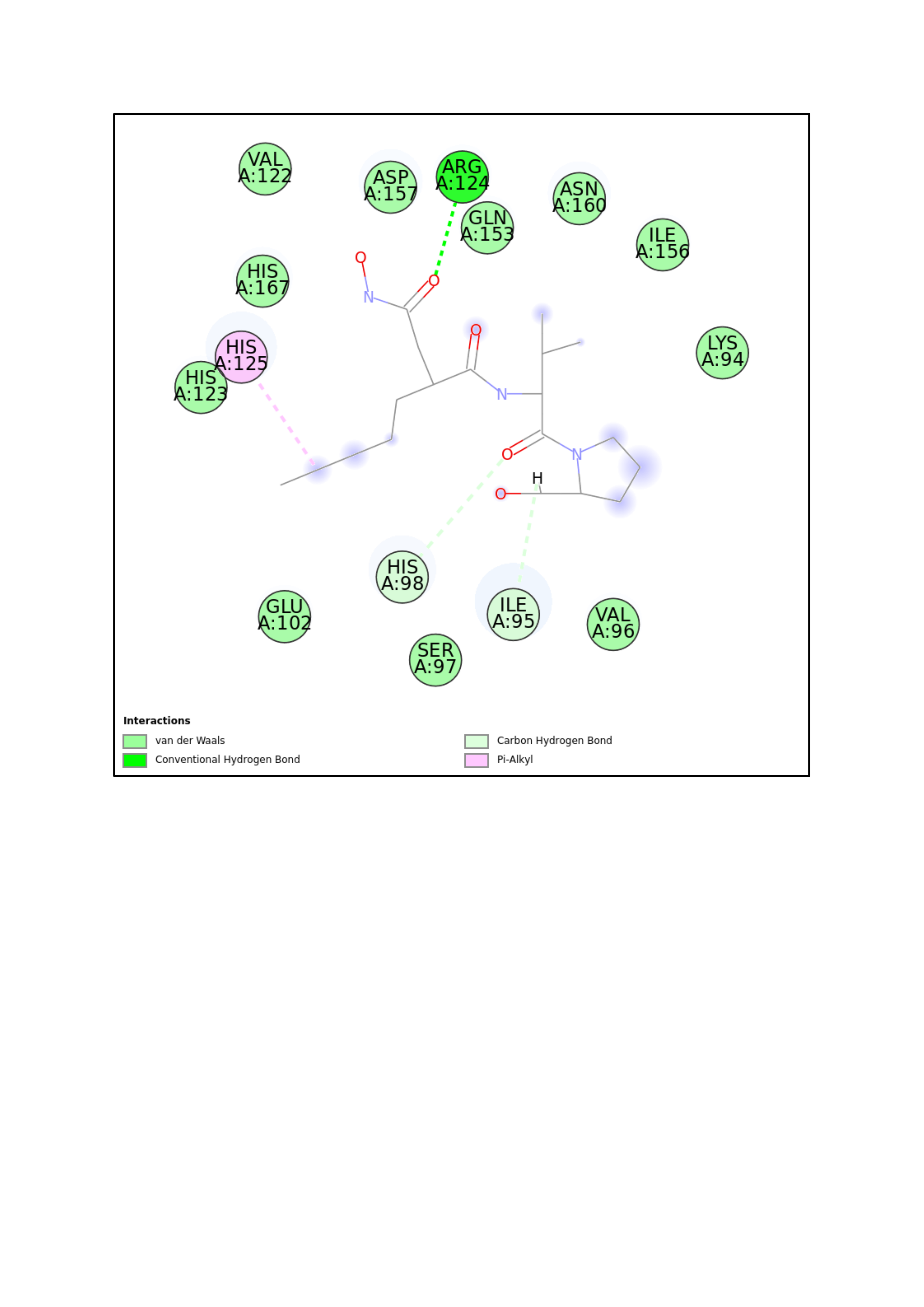

Supplement: S4 Fig — (TIF) [file pone.0315663.s004.tif]

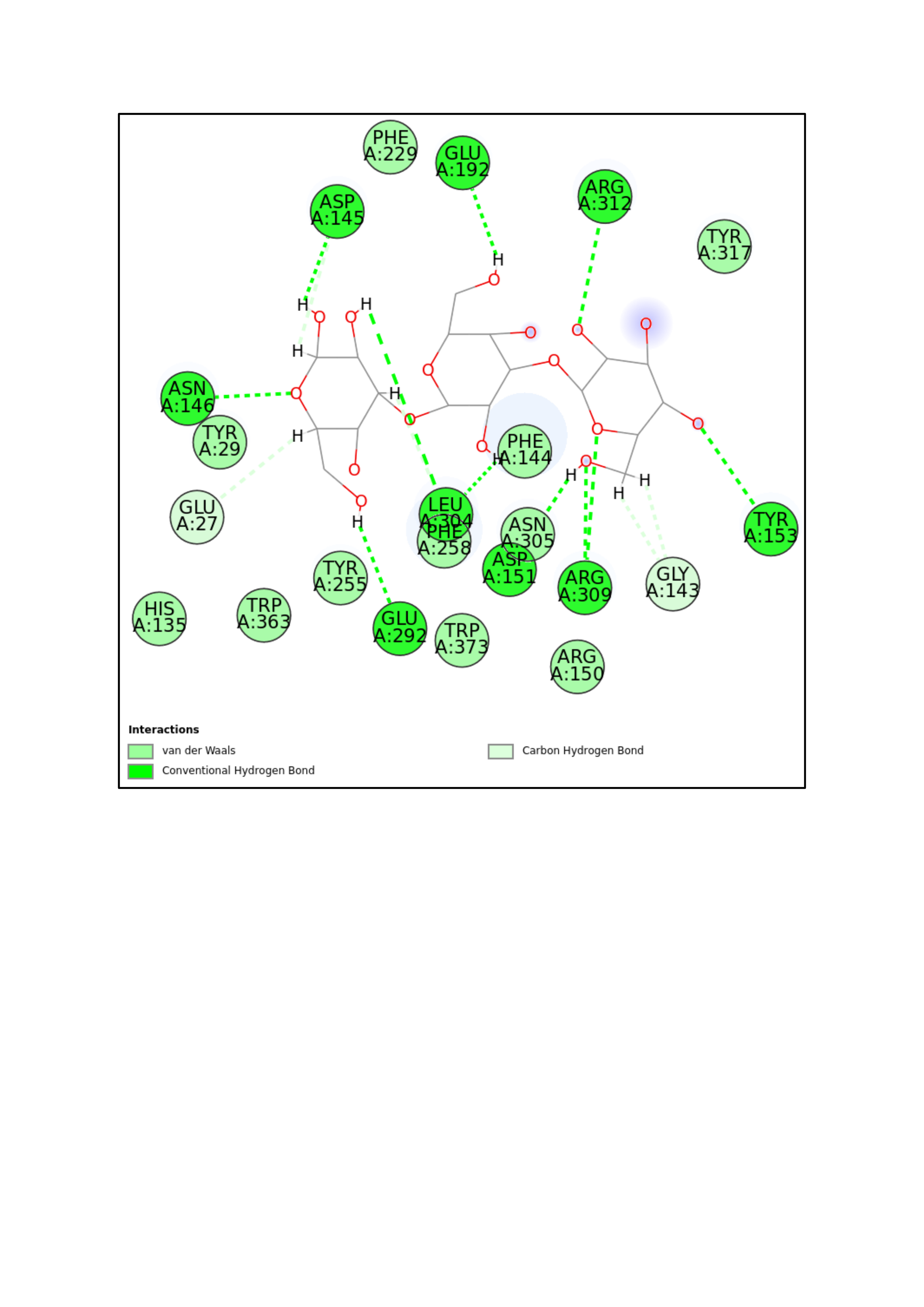

Supplement: S5 Fig — (TIF) [file pone.0315663.s005.tif]
