# Supplementary material for: Exploring the antimicrobial and antibiofilm potency of four essential oils against selected human pathogens using in vitro and in silico approaches
Source: PLoS One. 2025 Apr 24;20(4):e0315663. doi: 10.1371/journal.pone.0315663 (PMC12083874; doi:10.1371/journal.pone.0315663)
Supplement: S1 Table — (PDF) [file pone.0315663.s006.pdf]

S1 Table. Proteins (enzymes) selected for *in silico* molecular docking studies of active ingredients from CBO, BSO, CNBO, and CTLO targeting bacterial and fungal pathways.

#	Enzyme	Source Organism	FASTA Sequence	Amino Acid Length	PDB Id/Model	Pathway Involved
1.	Serine Protease (SplE)	*S. aureus*	GSEHNVKLIKNTNVAPYNGVVSIGSGTGFIV GKNTIVTNKHVVAGMEIGAHIIAHPNGEYN NGGFYKVKKIVRYSGQEDIAILHVEDKAVH PKNRNFKDYTGILKIASEAKENERISIVGYPE PYINKFQMYESTGKVLSVKGNMIITDAFVEP GNSGSAVFNSKYEVVGVHFGGNGPGNKSTK GYGVYFSPEIKKFIADNTDK	204	5MM8	Virulence factor
2.	β-Lactamase	*S. aureus*	KELNDLEKKYNAHIGVYALDTKSGKEVKFN SDKRFAYASTSKAINSAILLEQVPYNKLNKK VHINKDDIVAYSPILEKYVGKDITLKALIEAS MTYSDNTANNKIIKEIGGIKKVKQRLKELGD KVTNPVRYEIELNYYSPKSKKDTSTPAAFGK TLNKLIANGKLSKENKKFLLDLMLNNKSGD TLIKDGVPKDYKVADKSGQAITYASRNDVA FVYPKGQSEPIVLVIFTNKDNKSDKPNDKLIS ETAKSVMKEF	257	3BLM	β-Lactam antibiotics resistance
3.	Peptide Deformylase	*S. aureus*	MLTMKDIIRDGHPTLRQKAAELELPLTKEEK ETLIAMREFLVNSQDEEIAKRYGLRSGVGLA APQINISKRMIAVLIPDDGSGKSYDYMLVNP KIVSHSVQEAYLPTGEGCLSVDDNVAGLVH RHNKITIKAKDIEGNDIQLRLKGYPAIVFQHE IDHLNGVMFYDHIDKDHPLQPHTDAVEVH	184	1LMH	Protein synthesis
4.	Thymidilate Kinase (TMK)	*S. aureus*	MSAFITFEGPEGSGKTTVINEVYHRLVKDYD VIMTREPGGVPTGEEIRKIVLEGNDMDIRTE AMLFAASRREHLVLKVIPALKEGKVVLCDR YIDSSLAYQGYARGIGVEEVRALNEFAINGL YPDLTIYLNVSAEVGRERIIKNSRDQNRLDQ	205	4QGH	DNA biosynthesis

			EDLKFHEKVIEGYQEIIHNESQRFKSVNADQ PLENVVEDTYQTIIKYLEKI			
5.	Dihydrofolate Reductase (DHFR)	*S. aureus*	TLSILVAHDLQRVIGFENQLPWHLPNDLKHV KKLSTGHTLVMGRKTFESIGKPLPNRRNVVL TSDTSFNVEGVDVIHSIEDIYQLPGHVFIFGG QTLYEEMIDKVDDMYITVIEGKFRGDTFFPP YTFEDWEVASSVEGKLDEKNTIPHTFLHLIR K	157	3F0U	Folate metabolism, DNA synthesis and methylation
6.	Clumping Factor A (ClfA)	*S. aureus*	MRGSHHHHHHGSLVPRGSMVAADAPAAGT DITNQLTNVTVGIDSGTTVYPHQAGYVKLN YGFSVPNSAVKGDTFKITVPKELNLNGVTST AKVPPIMAGDQVLANGVIDSDGNVIYTFTD YVNTKDDVKATLTMPAYIDPENVKKTGNV TLATGIGSTTANKTVLVDYEKYGKFYNLSIK GTIDQIDKTNNTYRQTIYVNPSGDNVIAPVL TGNLKPNTDSNALIDQQNTSIKVYKVDNAA DLSESYFVNPENFEDVTNSVNITFPNPNQYK VEFNTPDDQITTPYIVVVNGHIDPNSKGDLA LRSTLYGYNSNIIWRSMSWDNEVAFNNGSG SGDGIDKPVVPEQPDEPGEIEPIPEK	359	1N67	Virulence factor
7.	Lipoprotein Signal Peptidase-II (LspA)	*S. aureus*	MGHHHHHHDYDIPTTENLYFQGAHMHKKY FIGTSILIAVFVVIFDQVTKYIIATTMKIGDSF EVIPHFLNITSHRNNGAAWGILSGKMTFFFII TIIILIALVYFFIKDAQYNLFMQVAISLLFAGA LGNFIDRVLTGEVVDFIDTNIFGYDFPIFNIAD SSLTIGVILIIIALLKDTSNKKEKEVK	187	6RYO	Virulence factor, lipoprotein synthesis in cell membrane
8.	Surface Protein-G (SasG)	*S. aureus*	GPHMIAPGHRDEFDPKLPTGEKEEVPGKPGI KNPETGDVVRPPVDSVTKYGPVKGDSIVEK EEIPFEKERKFNPDLAPGTEKVTREGQKGEK TITTPTLKNPLTGEIISKGESKEEITKDPINELT EWGPET	132	5DBL	Virulence factor, colonization, biofilm formation

9.	RNA Polymerase Sigma Factor rpoD	*S. aureus*	MKEQLEDVLDTLTDREENVLRLRFGLDDGRTRTLEEVGKVFGVTRERIRQIEAKALRKLRHPSRSKRLKDFMD	73	4G6D	Transcription
10.	Sortase A	*S. aureus*	MQAKPQIPKDKSKVAGYIEIPDADIKEPVYPGPATPEQLNRGVSFAEENESLDDQNISIAGHTFIDRPNYQFTNLKAAKKGSMVYFKVGNETRKYKMTSIRDVKPTDVEVLDEQKGKDKQLTLITCDDYNEKTGVWEKRKIFVATEVK	148	2MLM	Virulence factor
11.	DNA Topoisomerase IV, B Subunit	*S. aureus*	MAMNKQNNYSDDSIQVLEGLEAVRKRPGMYIGSTDKRGLHHLVYEIVDNSVDEVLNGYGNEIDVTINKDGSISIEDNGRGMPTGIHKSGKPTVEVIFTVLHAGGKFGQGGYKTSGGLHGVGASVVNALSEWLEVEIHRDGNIYHQSFKNGGSPSSGLVKKGKTKKTGTKVTFKPDDTIFKASTSFNFDVLSERLQESAFLLKNLKITLNDLRSGKERQEHYHYEE	225	4URN	DNA synthesis
12.	Outer Membrane Protein Assembly Factor BamA	*E. coli*	HMRNTGSFNFGIGYGTESGVSFQAGVQQDNWLGTGYAVGINGTKNDYQTYAELSVTNPYFTVDGVSLGGRLFYNDFQADDADLSDYTNKSYGTDVTLGFPINEYNSLRAGLGYVHNSLSNMQPQVAMWRYLYSMGEHPSTSDQDNSFKTDDFTFNYGWTYNKLDRGYFPTDGSRVNLTGKVTIPGSDNEYYKVTLDTATYVPIDDDHKWVVLGRTRWGYGDGLGGKEMPFYENFYAGGSSTVRGFQSNTIGPKAVYFPHQASNYDPDYDYESATQDGAKDLSKSDDAVGGNAMAVASLEFITPTPFISDKYANSVRTSFFWDMGTVWDTNWDSSQYSGYPDYSDPSNIRMSAGIALQWMSPLGPLVFSYAQPFKKYDGDKAEQFQFNIGKTW	392	7R1V	OMP assembly
13.	Macrolide 2'-phosphotransferase I	*E. coli*	MTVVTTADTSQLYALAARHGLKLHGPLTVNELGLDYRIVIATVDDGRRWVLRIPRRAEVSAKVEPEARVLAMLKNRLPFAVPDWRVANA	301	5IGH	Macrolide antibiotic degradation

			ELVAYPMLEDSTAMVIQPGSSTPDWVVPQD SEVFAESFATALAALHAVPISAAVDAGMLIR TPTQARQKVADDVDRVRREFVVNDKRLHR WQRWLDDDSSWPDFSVVVHGDLYVGHVLI DNTERVSGMIDWSEARVDDPAIDMAAHLM VFGEEGLAKLLLTYEAAGGRVWPRLAHHIA ERLAFGAVTYALFALDSGNEEYLAAAKAQL AAAE			
14.	*E. coli* Rhomboid Protease GlpG	*E. coli*	ERAGPVTWVMMIACVVVFIAMQILGDQEV MLWLAWPFDPTLKFEFWRYFTHALMHFSL MHILFNLLWWWYLGGAVEKRLGSGKLIVIT LISALLSGYVQQKFSGPWFGGLSGVVYALM GYVWLRGERDPQSGIYLQRGLIIFALIWIVA GWFDLFGMSMANGAHIAGLAVGLAMAFVD SL	180	3B45	Catalyzes intramembrane proteolysis
15.	Secreted Aspartic Proteinase	*C. albicans*	QAVPVTLHNEQVTYAADITVGSNNQKLNVI VDTGSSDLWVPDVNIDCQVTYSDQTADFCK QKGTYDPSGSSASQDLNTPFSIGYGDGSSSQ GTLYKDTVGFGGVSIKNQVLADVDSTSIDQ GILGVGYKTNEAGGSYDNVPVTLKKQGVIA KNAYSLYLNSPDSATGQIIFGGVDNAKYSGS LIALPVTSDRELRISLGSVEVSGKTINTDNVD VLLDSGTTITYLQQDLADQIIKAFNGKLTQD SNGNSFYEVDCNLSGDVVFNFSKNAKISVPA SDFAASTQGDDGQPYDKCQLLFDVNKANIL GDNFLRSAYIVYDLDDNEISIAQVKYTSASS TSALT	342	1ZAP	Virulence factor
16.	*C. albicans* Dihydrofolate Reductase (CaDHFR)	*C. albicans*	MLKPNVAIIVAALKPALGIGYKGKMPWRLR KEIRYFKDVTTRTTKPNTRNAVIMGRKTWE SIPQKFRPLPDRLNIILSRSYENEIIDDNIIHAS SIESSLNLVSDVERVFIIGGAEIYNELINNSLV SHLLITEIEHPSPESIEMDTFLKFPLESWTKQP	192	3QLS	Folate metabolism, DNA synthesis

			KSELQKFVGDTVLEDDIKEGDFTYNYTLWT RK			
17.	Acetohydroxyacid Synthase (AHAS)	*C. albicans*	MHHHHHHSSGLVPRGSGMKETAAAKFERQ HMDSPDLGTDDDDKAMAFNTADTSTQPIIN DPTLNKHQSSAISRKKKEQLMDDSFIGLTGG EIFHEMMLRHKVDTVFGYAGGAILPVFDAIY NSDKFKFVLPRHEQGAGHMAEGYARASGK PGVVLVTSGPGATNVITPMADALMDGVPLV VFSGQVPTTAIGTDAFQEADIVGISRSCTKW NVMVKNVAELPRRINEAFEIATTGRPGPVLV DLPKDVTASILRESIPINTTLPSNALSQITKKA VSEFTSEAIKRAANILNKAKKPIIYAGAGILN NEQGPKLLKELADKANIPVTTTLQGLGAFD QRDPKSLDMLGMHGSAAANTAIQNADCIIA LGARFDDRVTGNISKFAPEAKLAASEGRGGI LHFEISPKNINKVVEATEAIEGDVTANLQSFI PLVDSIENRPEWFNKINEWKKKYPYSYQLET PGSLIKPQTLIKEISDQAQTYNKEVIVTTGVG QHQMWAAQHFTWTQPRTMITSGGLGTMGY GLPAAIGAQVAKPDAIVIDIDGDASFNMTLT ELSSAVQAGAPIKVCVLNNEEQGMVTQWQS LFYEHRYSHTHQSNPDFMKLAESMNVKGIRI TNQQELKSGVKEFLDATEPVLLEVIVEKKVP VLPMVPAGKALDDFILWDAEVEKQQNDLR KERTGGKY	682	6DEN	Branched-chain amino acid (BCAA) biosynthesis pathway
18.	Serine/Threonine Phosphatase Z1 (PPZ1)	*C. albicans*	GHMIDIDSLIDKLLNAGFSGKRTKNVCLKNT EIELICASAREIFLSQPSLLELAPPVKVVGDV HGQYHDLIRIFSKCGFPPKTNYLFLGDYVNR GKQSLETILLLLCYKIKYPENFFLLRGNHECA NVTRVYGFYDECKRRCNIKTWKLFIDTFNTL PIAAIVAGKIFCVHGGLSPVLNSMDEIRNIAR PTDVPDFGLLNDLLWSDPADTINEWEDNER GVSYVFSKVAINKFLSKFNFDLVCRAHMVV	317	5JPF	Virulence factor, protection from oxidative damage of the cell membrane

			EDGYEFFNDRTLVTVFSAPNYCGEFDNWGA VMGVSEDLLCSFELLDPLDSAALKQVMKKE KQERKKST			
19.	Exo-β-(1,3)-Glucanase	*C. albicans*	AWDYDNNVIRGVNLGGWFVLEPYMTPSLF EPFQNGNDQSGVPVDEYHWTQTLGKEAAS RILQKHWSTWITEQDFKQISNLGLNFVRIPIG YWAFQLLDNDPYVQGQVQYLEKALGWAR KNNIRVWIDLHGAPGSQNGFDNSGLRDSYN FQNGDNTQVTLNVLNTIFKKYGGNEYSDVV IGIELLNEPLGPVLNMDKLKQFFLDGYNSLR QTGSVTPVIIHDAFQVFGYWNNFLTVAEGQ WNVVVDHHHYQVFSGGELSRNINDHISVAC NWGWDAKKESHWNVAGEWSAALTDCAK WLNGVNRGARYEGAYDNAPYIGSCQPLLDI SQWSDEHKTDTRRYIEAQLDAFEYTGGWVF WSWKTENAPEWSFQTLTYNGLFPQPVTDRQ FPNQCGFH	394	1EQP	Cell wall remodeling (germination of conidia)
20.	Delta(14)-Sterol Reductase	*C. albicans* (strain WO-1)	MKSSKLNPVTTHKEFNGISGALGITIGLPTLT VLFYLLCNQTYSIHGINVDFAKIKSQLPITQD ELWQLVFDKTCWSAYLAWFFILVILDYLLP GKSLNGVKLRDGTVLNYKINGLSMSSLLIVL LLARLFQSNSDSSLEYYLPELQFIYDNQLQLI IICFLFSFMLAVFVYIISFIPLAKPNGIGTKERI LSINGNTGNPFYDWFIGRELNPRIGSWDIKLF CELRPGMLLWLLINLSCLHYQYHNLGYVTD SMIVVNLLQAFYIFDGVLNEEGCLTMIDITT DGFGFMLSFGDLAWVPWTYSLQARYLSIKG NEVNLGWTLSLLIVGLQALGFYIFRSANKQK SDFRQGKLPHLKSIQTKTGSKLLVEGWWGL SQHINYLGDWLIGLSWCLPTGFQTPLTYFYV IYFASLLIHRQVRDEMKCRAKYGEDWEKYE KLVPYKIIPYVY	448	AlphaFold model: Confidence score_very high (pLDDT > 90)	Sterol biosynthesis

21.	Thymidylate Kinase	*C. albicans*	GSTSARGQLILIEGLDRSGKSTQASILSTKLSP<br>SKLIKFPDRSTPIGKLINEYLTNKSFTLSDQA<br>AHLLFSANRWELSQQIQDLLNQGYFIILDRYI<br>YSGIAYTLAKNDFHDETISQGKNKQQLNNID<br>WLLSPDKGLPKPDLTLFLTLDLEEISKRKGW<br>GDERYELQQFQAKVKQCFLEILDTNKDPTIR<br>IVDVGGKTIDQVTTQLWEIIETNKNHELINDS<br>IQFIT	227	5UIV	Biosynthesis of thymidine triphosphate (dTTP)
22.	N-Myristoyl Transferase (NMT)	*A. fumigatus*	GPRSQTQPVPRFDETSTDTGGPIKIIDPEKVS<br>KEPDALLEGFEWATLDLTNETELQELWDLL<br>TYHYVEDDNAMFRFRYSQSFLHWALMSPG<br>WKKEWHVGVRATKSRKLVASICGVPTEINV<br>RNQKLKVVEINFLCIHKKLRSKRLTPVLIKEI<br>TRRCYLNGIYQAIYTAGVVLPTPVSSCRYYH<br>RPLDWLKLYEVGFSPLPAGSTKARQITKNHL<br>PSTTSTPGLRPMEPKDIDTVHDLLQRYLSRF<br>ALNQAFTREEVDHWLVHKPETVKEQVVWA<br>YVVEDPETHKITDFFSFYNLESTVIQNPKHD<br>NVRAAYLYYYATETAFTNNMKALKERLLM<br>LMNDALILAKKAHFDVFNALTLHDNPLFLE<br>QLKFGAGDGQLHFYLYNYRTAPVPGGVNE<br>KNLPDEKRMGGVGIVML	411	4CAV	Cell wall synthesis
23.	*A. flavus* Squalene Synthase (AfSQS)	*A. flavus*	MRATEVLYYMLRPSQLRSIVQWKVWHNPV<br>HERNVNNETETQKACFKFLDLTSRSFSAVIK<br>ELHPELLLPVCVFYLVLRGLDTIEDDTSIPLK<br>TKEPMLREFKDYLEQDGWTFDGNRPEEKDR<br>ELLVQFHNVITEFKNMKPAYREIVKDITDKM<br>GNGMADYCRKAEFEDASVKTIEEYDLYCYY<br>VAGLVGEGLTRLFVEAEFGNPALLSRPRLHK<br>SMGLFLQKTNIIRDVREDHDDDRHFWPKEI<br>WSKYVTEFEDLFKPENRETALNCGSEMVLN<br>ALEHAEECLFYLAGLREQSVFNFCAIPQAMA<br>IATLELCFRNPDMFDRNIKITKGEACQLMME	470	7WGH	Ergosterol biosynthesis

			STQNLHVLCDTFRRYARRIHKKNTPKDPNFLKISIVCGKIEKFIDTIFPQQTAAQAKLKVQGEKSEAEKEKARQEAETRQDLYFMLALMGVIVLIVSIIMLTAAWLLGARFDLAFQELKSGNFRPPAKQIPGEL			

Note: ***S. aureus*** = *Staphylococcus aureus*; **MRSA** = Methicillin-resistant *Staphylococcus aureus*; ***E. coli*** = *Escherichia coli*; ***P. aeruginosa*** = *Pseudomonas aeruginosa*; ***S. typhi*** = *Salmonella typhi*; ***P. mirabilis*** = *Proteus mirabilis*; ***C. albicans*** = *Candida albicans*; ***A. fumigatus*** = *Aspergillus fumigatus*; ***A. flavus*** = *Aspergillus flavus*.
