# Supplementary material for: Exploring the antimicrobial and antibiofilm potency of four essential oils against selected human pathogens using in vitro and in silico approaches
Source: PLoS One. 2025 Apr 24;20(4):e0315663. doi: 10.1371/journal.pone.0315663 (PMC12083874; doi:10.1371/journal.pone.0315663)
Supplement: S2 Table — (PDF) [file pone.0315663.s007.pdf]

S2 Table. One-way ANOVA of antimicrobial activity of essential oils against tested microorganisms.

ANOVA						
		**Sum of Squares**	**df**	**Mean Square**	**F**	**Sig.**
CBO	Between Groups	1501.895	18	83.439	1042.982	.000
	Within Groups	3.040	38	.080		
	Total	1504.935	56			
BSO	Between Groups	8771.053	18	487.281	8652.648	.000
	Within Groups	2.140	38	.056		
	Total	8773.193	56			
CNBO	Between Groups	3489.474	18	193.860	1796.748	.000
	Within Groups	4.100	38	.108		
	Total	3493.574	56			
CTLO	Between Groups	146.211	18	8.123	1028.889	.000
	Within Groups	.300	38	.008		
	Total	146.511	56			
