# Supplementary material for: Exploring the antimicrobial and antibiofilm potency of four essential oils against selected human pathogens using in vitro and in silico approaches
Source: PLoS One. 2025 Apr 24;20(4):e0315663. doi: 10.1371/journal.pone.0315663 (PMC12083874; doi:10.1371/journal.pone.0315663)
Supplement: S3 Table — (PDF) [file pone.0315663.s008.pdf]

S3 Table. Post Hoc analysis of antimicrobial activity of essential oils against tested microorganisms.

Post Hoc Tests							
**Multiple Comparisons**							
**Tukey HSD**							
**Dependent Variable**	**(I) Volatile oils**	**(J) Volatile oils**	**Mean Difference (I-J)**	**Std. Error**	**Sig.**	**95% Confidence Interval**	
						**Lower Bound**	**Upper Bound**
S. aureus ATCC 29213	CBO	BSO	-25.0000*	.2160	.000	-25.692	-24.308
		CNBO	-13.0000*	.2160	.000	-13.692	-12.308
		CTLO	11.0000*	.2160	.000	10.308	11.692
	BSO	CBO	25.0000*	.2160	.000	24.308	25.692
		CNBO	12.0000*	.2160	.000	11.308	12.692
		CTLO	36.0000*	.2160	.000	35.308	36.692
	CNBO	CBO	13.0000*	.2160	.000	12.308	13.692
		BSO	-12.0000*	.2160	.000	-12.692	-11.308
		CTLO	24.0000*	.2160	.000	23.308	24.692
	CTLO	CBO	-11.0000*	.2160	.000	-11.692	-10.308
		BSO	-36.0000*	.2160	.000	-36.692	-35.308
		CNBO	-24.0000*	.2160	.000	-24.692	-23.308
S. aureus-CI	CBO	BSO	-5.0000*	.1915	.000	-5.613	-4.387
		CNBO	-8.0000*	.1915	.000	-8.613	-7.387
		CTLO	8.0000*	.1915	.000	7.387	8.613
	BSO	CBO	5.0000*	.1915	.000	4.387	5.613
		CNBO	-3.0000*	.1915	.000	-3.613	-2.387
		CTLO	13.0000*	.1915	.000	12.387	13.613
	CNBO	CBO	8.0000*	.1915	.000	7.387	8.613
		BSO	3.0000*	.1915	.000	2.387	3.613

		CTLO	16.0000*	.1915	.000	15.387	16.613
	CTLO	CBO	-8.0000*	.1915	.000	-8.613	-7.387
		BSO	-13.0000*	.1915	.000	-13.613	-12.387
		CNBO	-16.0000*	.1915	.000	-16.613	-15.387
MRSA-1	CBO	BSO	-17.0000*	.2082	.000	-17.667	-16.333
		CNBO	-13.0000*	.2082	.000	-13.667	-12.333
		CTLO	6.0000*	.2082	.000	5.333	6.667
	BSO	CBO	17.0000*	.2082	.000	16.333	17.667
		CNBO	4.0000*	.2082	.000	3.333	4.667
		CTLO	23.0000*	.2082	.000	22.333	23.667
	CNBO	CBO	13.0000*	.2082	.000	12.333	13.667
		BSO	-4.0000*	.2082	.000	-4.667	-3.333
		CTLO	19.0000*	.2082	.000	18.333	19.667
	CTLO	CBO	-6.0000*	.2082	.000	-6.667	-5.333
		BSO	-23.0000*	.2082	.000	-23.667	-22.333
		CNBO	-19.0000*	.2082	.000	-19.667	-18.333
MRSA-2	CBO	BSO	-8.0000*	.1915	.000	-8.613	-7.387
		CNBO	-8.0000*	.1915	.000	-8.613	-7.387
		CTLO	6.0000*	.1915	.000	5.387	6.613
	BSO	CBO	8.0000*	.1915	.000	7.387	8.613
		CNBO	.0000	.1915	1.000	-.613	.613
		CTLO	14.0000*	.1915	.000	13.387	14.613
	CNBO	CBO	8.0000*	.1915	.000	7.387	8.613
		BSO	.0000	.1915	1.000	-.613	.613
		CTLO	14.0000*	.1915	.000	13.387	14.613
	CTLO	CBO	-6.0000*	.1915	.000	-6.613	-5.387
		BSO	-14.0000*	.1915	.000	-14.613	-13.387

		CNBO	-14.0000*	.1915	.000	-14.613	-13.387
S. saprophyticus ATCC 43867	CBO	BSO	-21.0000*	.1915	.000	-21.613	-20.387
		CNBO	-13.0000*	.1915	.000	-13.613	-12.387
		CTLO	9.0000*	.1915	.000	8.387	9.613
	BSO	CBO	21.0000*	.1915	.000	20.387	21.613
		CNBO	8.0000*	.1915	.000	7.387	8.613
		CTLO	30.0000*	.1915	.000	29.387	30.613
	CNBO	CBO	13.0000*	.1915	.000	12.387	13.613
		BSO	-8.0000*	.1915	.000	-8.613	-7.387
		CTLO	22.0000*	.1915	.000	21.387	22.613
	CTLO	CBO	-9.0000*	.1915	.000	-9.613	-8.387
		BSO	-30.0000*	.1915	.000	-30.613	-29.387
		CNBO	-22.0000*	.1915	.000	-22.613	-21.387
S. epidermidis ATCC 12228	CBO	BSO	-26.0000*	.1414	.000	-26.453	-25.547
		CNBO	-12.0000*	.1414	.000	-12.453	-11.547
		CTLO	14.0000*	.1414	.000	13.547	14.453
	BSO	CBO	26.0000*	.1414	.000	25.547	26.453
		CNBO	14.0000*	.1414	.000	13.547	14.453
		CTLO	40.0000*	.1414	.000	39.547	40.453
	CNBO	CBO	12.0000*	.1414	.000	11.547	12.453
		BSO	-14.0000*	.1414	.000	-14.453	-13.547
		CTLO	26.0000*	.1414	.000	25.547	26.453
	CTLO	CBO	-14.0000*	.1414	.000	-14.453	-13.547
		BSO	-40.0000*	.1414	.000	-40.453	-39.547
		CNBO	-26.0000*	.1414	.000	-26.453	-25.547
S. pyogenes (A) ATCC 19615	CBO	BSO	-6.0000*	.2160	.000	-6.692	-5.308
		CNBO	-8.0000*	.2160	.000	-8.692	-7.308

		CTLO	5.0000*	.2160	.000	4.308	5.692
	BSO	CBO	6.0000*	.2160	.000	5.308	6.692
		CNBO	-2.0000*	.2160	.000	-2.692	-1.308
		CTLO	11.0000*	.2160	.000	10.308	11.692
	CNBO	CBO	8.0000*	.2160	.000	7.308	8.692
		BSO	2.0000*	.2160	.000	1.308	2.692
		CTLO	13.0000*	.2160	.000	12.308	13.692
	CTLO	CBO	-5.0000*	.2160	.000	-5.692	-4.308
		BSO	-11.0000*	.2160	.000	-11.692	-10.308
		CNBO	-13.0000*	.2160	.000	-13.692	-12.308
S. pneumoniae ATCC 49619	CBO	BSO	-7.00000*	.19579	.000	-7.6270	-6.3730
		CNBO	-8.00000*	.19579	.000	-8.6270	-7.3730
		CTLO	6.00000*	.19579	.000	5.3730	6.6270
	BSO	CBO	7.00000*	.19579	.000	6.3730	7.6270
		CNBO	-1.00000*	.19579	.004	-1.6270	-.3730
		CTLO	13.00000*	.19579	.000	12.3730	13.6270
	CNBO	CBO	8.00000*	.19579	.000	7.3730	8.6270
		BSO	1.00000*	.19579	.004	.3730	1.6270
		CTLO	14.00000*	.19579	.000	13.3730	14.6270
	CTLO	CBO	-6.00000*	.19579	.000	-6.6270	-5.3730
		BSO	-13.00000*	.19579	.000	-13.6270	-12.3730
		CNBO	-14.00000*	.19579	.000	-14.6270	-13.3730
E. faecalis ATCC 29212	CBO	BSO	-7.00000*	.17321	.000	-7.5547	-6.4453
		CNBO	-8.00000*	.17321	.000	-8.5547	-7.4453
		CTLO	6.00000*	.17321	.000	5.4453	6.5547
	BSO	CBO	7.00000*	.17321	.000	6.4453	7.5547
		CNBO	-1.00000*	.17321	.002	-1.5547	-.4453

		CTLO	13.00000*	.17321	.000	12.4453	13.5547
	CNBO	CBO	8.00000*	.17321	.000	7.4453	8.5547
		BSO	1.00000*	.17321	.002	.4453	1.5547
		CTLO	14.00000*	.17321	.000	13.4453	14.5547
	CTLO	CBO	-6.00000*	.17321	.000	-6.5547	-5.4453
		BSO	-13.00000*	.17321	.000	-13.5547	-12.4453
		CNBO	-14.00000*	.17321	.000	-14.5547	-13.4453
B. cereus ATCC 10876	CBO	BSO	-7.00000*	.29155	.000	-7.9336	-6.0664
		CNBO	-9.00000*	.29155	.000	-9.9336	-8.0664
		CTLO	5.00000*	.29155	.000	4.0664	5.9336
	BSO	CBO	7.00000*	.29155	.000	6.0664	7.9336
		CNBO	-2.00000*	.29155	.001	-2.9336	-1.0664
		CTLO	12.00000*	.29155	.000	11.0664	12.9336
	CNBO	CBO	9.00000*	.29155	.000	8.0664	9.9336
		BSO	2.00000*	.29155	.001	1.0664	2.9336
		CTLO	14.00000*	.29155	.000	13.0664	14.9336
	CTLO	CBO	-5.00000*	.29155	.000	-5.9336	-4.0664
		BSO	-12.00000*	.29155	.000	-12.9336	-11.0664
		CNBO	-14.00000*	.29155	.000	-14.9336	-13.0664
E. coli ATCC 25922	CBO	BSO	8.00000*	.12910	.000	7.5866	8.4134
		CNBO	-5.00000*	.12910	.000	-5.4134	-4.5866
		CTLO	8.00000*	.12910	.000	7.5866	8.4134
	BSO	CBO	-8.00000*	.12910	.000	-8.4134	-7.5866
		CNBO	-13.00000*	.12910	.000	-13.4134	-12.5866
		CTLO	.00000	.12910	1.000	-.4134	.4134
	CNBO	CBO	5.00000*	.12910	.000	4.5866	5.4134
		BSO	13.00000*	.12910	.000	12.5866	13.4134

		CTLO	13.00000*	.12910	.000	12.5866	13.4134
	CTLO	CBO	-8.00000*	.12910	.000	-8.4134	-7.5866
		BSO	.00000	.12910	1.000	-.4134	.4134
		CNBO	-13.00000*	.12910	.000	-13.4134	-12.5866
K. pneumoniae ATCC 27736	CBO	BSO	9.00000*	.17795	.000	8.4301	9.5699
		CNBO	-3.00000*	.17795	.000	-3.5699	-2.4301
		CTLO	9.00000*	.17795	.000	8.4301	9.5699
	BSO	CBO	-9.00000*	.17795	.000	-9.5699	-8.4301
		CNBO	-12.00000*	.17795	.000	-12.5699	-11.4301
		CTLO	.00000	.17795	1.000	-.5699	.5699
	CNBO	CBO	3.00000*	.17795	.000	2.4301	3.5699
		BSO	12.00000*	.17795	.000	11.4301	12.5699
		CTLO	12.00000*	.17795	.000	11.4301	12.5699
	CTLO	CBO	-9.00000*	.17795	.000	-9.5699	-8.4301
		BSO	.00000	.17795	1.000	-.5699	.5699
		CNBO	-12.00000*	.17795	.000	-12.5699	-11.4301
P. aerugenosa ATCC 9027	CBO	BSO	3.00000*	.15811	.000	2.4937	3.5063
		CNBO	-4.00000*	.15811	.000	-4.5063	-3.4937
		CTLO	3.00000*	.15811	.000	2.4937	3.5063
	BSO	CBO	-3.00000*	.15811	.000	-3.5063	-2.4937
		CNBO	-7.00000*	.15811	.000	-7.5063	-6.4937
		CTLO	.00000	.15811	1.000	-.5063	.5063
	CNBO	CBO	4.00000*	.15811	.000	3.4937	4.5063
		BSO	7.00000*	.15811	.000	6.4937	7.5063
		CTLO	7.00000*	.15811	.000	6.4937	7.5063
	CTLO	CBO	-3.00000*	.15811	.000	-3.5063	-2.4937
		BSO	.00000	.15811	1.000	-.5063	.5063

		CNBO	-7.00000*	.15811	.000	-7.5063	-6.4937
S. typhimurium ATCC 13311	CBO	BSO	11.00000*	.17795	.000	10.4301	11.5699
		CNBO	-6.00000*	.17795	.000	-6.5699	-5.4301
		CTLO	11.00000*	.17795	.000	10.4301	11.5699
	BSO	CBO	-11.00000*	.17795	.000	-11.5699	-10.4301
		CNBO	-17.00000*	.17795	.000	-17.5699	-16.4301
		CTLO	.00000	.17795	1.000	-.5699	.5699
	CNBO	CBO	6.00000*	.17795	.000	5.4301	6.5699
		BSO	17.00000*	.17795	.000	16.4301	17.5699
		CTLO	17.00000*	.17795	.000	16.4301	17.5699
	CTLO	CBO	-11.00000*	.17795	.000	-11.5699	-10.4301
		BSO	.00000	.17795	1.000	-.5699	.5699
		CNBO	-17.00000*	.17795	.000	-17.5699	-16.4301
S. flexneri ATCC 12022	CBO	BSO	11.00000*	.22730	.000	10.2721	11.7279
		CNBO	-10.00000*	.22730	.000	-10.7279	-9.2721
		CTLO	11.00000*	.22730	.000	10.2721	11.7279
	BSO	CBO	-11.00000*	.22730	.000	-11.7279	-10.2721
		CNBO	-21.00000*	.22730	.000	-21.7279	-20.2721
		CTLO	.00000	.22730	1.000	-.7279	.7279
	CNBO	CBO	10.00000*	.22730	.000	9.2721	10.7279
		BSO	21.00000*	.22730	.000	20.2721	21.7279
		CTLO	21.00000*	.22730	.000	20.2721	21.7279
	CTLO	CBO	-11.00000*	.22730	.000	-11.7279	-10.2721
		BSO	.00000	.22730	1.000	-.7279	.7279
		CNBO	-21.00000*	.22730	.000	-21.7279	-20.2721
P. vulgaris ATCC 6380	CBO	BSO	11.00000*	.22730	.000	10.2721	11.7279
		CNBO	-9.00000*	.22730	.000	-9.7279	-8.2721

		CTLO	11.00000*	.22730	.000	10.2721	11.7279
	BSO	CBO	-11.00000*	.22730	.000	-11.7279	-10.2721
		CNBO	-20.00000*	.22730	.000	-20.7279	-19.2721
		CTLO	.00000	.22730	1.000	-.7279	.7279
	CNBO	CBO	9.00000*	.22730	.000	8.2721	9.7279
		BSO	20.00000*	.22730	.000	19.2721	20.7279
		CTLO	20.00000*	.22730	.000	19.2721	20.7279
	CTLO	CBO	-11.00000*	.22730	.000	-11.7279	-10.2721
		BSO	.00000	.22730	1.000	-.7279	.7279
		CNBO	-20.00000*	.22730	.000	-20.7279	-19.2721
P. mirabilis ATCC 29906	CBO	BSO	7.00000*	.15811	.000	6.4937	7.5063
		CNBO	-11.00000*	.15811	.000	-11.5063	-10.4937
		CTLO	7.00000*	.15811	.000	6.4937	7.5063
	BSO	CBO	-7.00000*	.15811	.000	-7.5063	-6.4937
		CNBO	-18.00000*	.15811	.000	-18.5063	-17.4937
		CTLO	.00000	.15811	1.000	-.5063	.5063
	CNBO	CBO	11.00000*	.15811	.000	10.4937	11.5063
		BSO	18.00000*	.15811	.000	17.4937	18.5063
		CTLO	18.00000*	.15811	.000	17.4937	18.5063
	CTLO	CBO	-7.00000*	.15811	.000	-7.5063	-6.4937
		BSO	.00000	.15811	1.000	-.5063	.5063
		CNBO	-18.00000*	.15811	.000	-18.5063	-17.4937
C. albicans ATCC 10231	CBO	BSO	13.00000*	.28284	.000	12.0942	13.9058
		CNBO	-26.00000*	.28284	.000	-26.9058	-25.0942
		CTLO	10.00000*	.28284	.000	9.0942	10.9058
	BSO	CBO	-13.00000*	.28284	.000	-13.9058	-12.0942
		CNBO	-39.00000*	.28284	.000	-39.9058	-38.0942

		CTLO	-3.00000*	.28284	.000	-3.9058	-2.0942
	CNBO	CBO	26.00000*	.28284	.000	25.0942	26.9058
		BSO	39.00000*	.28284	.000	38.0942	39.9058
		CTLO	36.00000*	.28284	.000	35.0942	36.9058
	CTLO	CBO	-10.00000*	.28284	.000	-10.9058	-9.0942
		BSO	3.00000*	.28284	.000	2.0942	3.9058
		CNBO	-36.00000*	.28284	.000	-36.9058	-35.0942
A. niger ATCC 6275	CBO	BSO	21.00000*	.25495	.000	20.1836	21.8164
		CNBO	-4.00000*	.25495	.000	-4.8164	-3.1836
		CTLO	24.00000*	.25495	.000	23.1836	24.8164
	BSO	CBO	-21.00000*	.25495	.000	-21.8164	-20.1836
		CNBO	-25.00000*	.25495	.000	-25.8164	-24.1836
		CTLO	3.00000*	.25495	.000	2.1836	3.8164
	CNBO	CBO	4.00000*	.25495	.000	3.1836	4.8164
		BSO	25.00000*	.25495	.000	24.1836	25.8164
		CTLO	28.00000*	.25495	.000	27.1836	28.8164
	CTLO	CBO	-24.00000*	.25495	.000	-24.8164	-23.1836
		BSO	-3.00000*	.25495	.000	-3.8164	-2.1836
		CNBO	-28.00000*	.25495	.000	-28.8164	-27.1836
*The mean difference is significant at the 0.05 level.							
