# Supplementary material for: Exploring the antimicrobial and antibiofilm potency of four essential oils against selected human pathogens using in vitro and in silico approaches
Source: PLoS One. 2025 Apr 24;20(4):e0315663. doi: 10.1371/journal.pone.0315663 (PMC12083874; doi:10.1371/journal.pone.0315663)
Supplement: S4 Table — (PDF) [file pone.0315663.s009.pdf]

S4 Table. The selected target proteins and their standard/co-crystallized ligands.

Target Protein_PDB Id	Standard or Co-crystallized Ligand
**Serine Protease (SplE)_5MM8**	**Benzamidine** 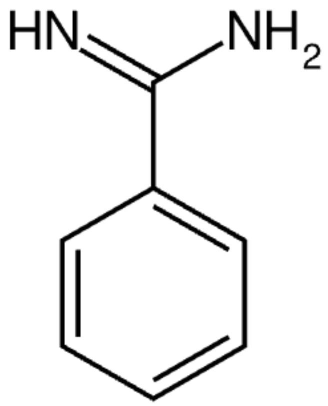
**Thymidylate Kinase_5UIV**	**Thymidine-5'-phosphate** 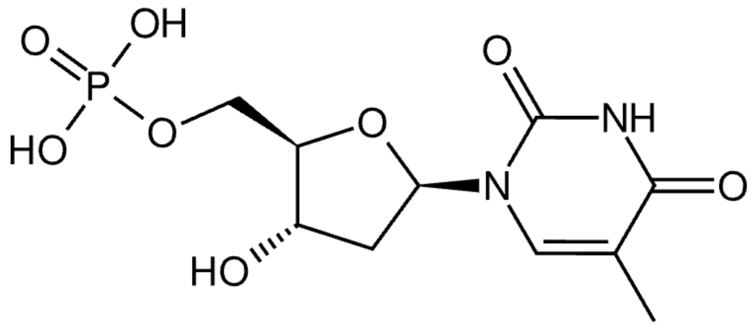

**Serine/Threonine Phosphatase Z1 (PPZ1)_5JPF**	**<u>Microcystin-LR</u>**
	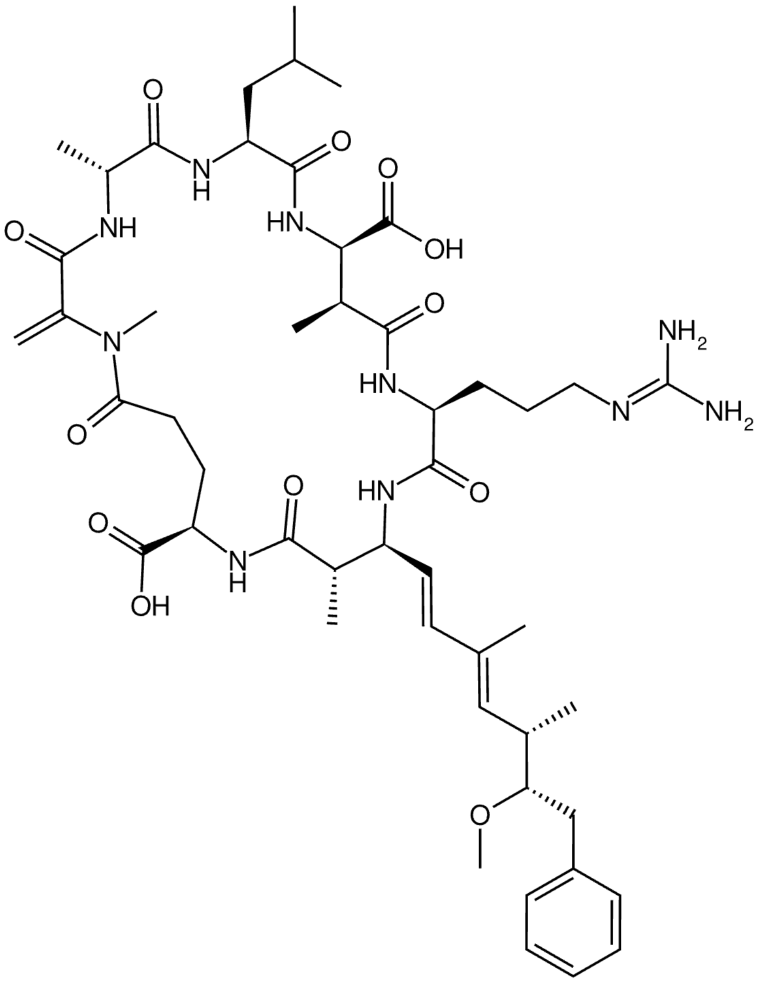

**Peptide Deformylase_1LMH**	**Actinonin**<br>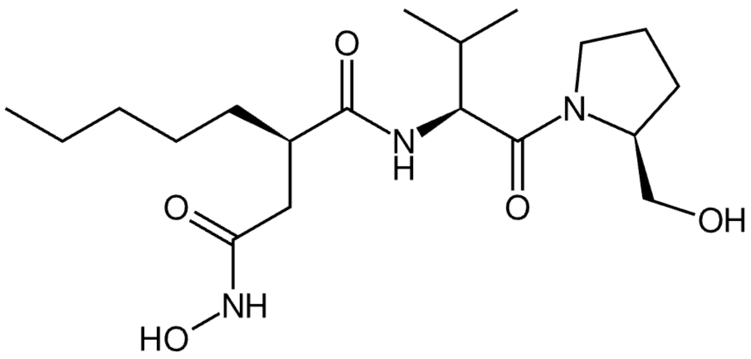
**Exo-β-(1,3)-Glucanase_1EQP**	**Laminaran**<br>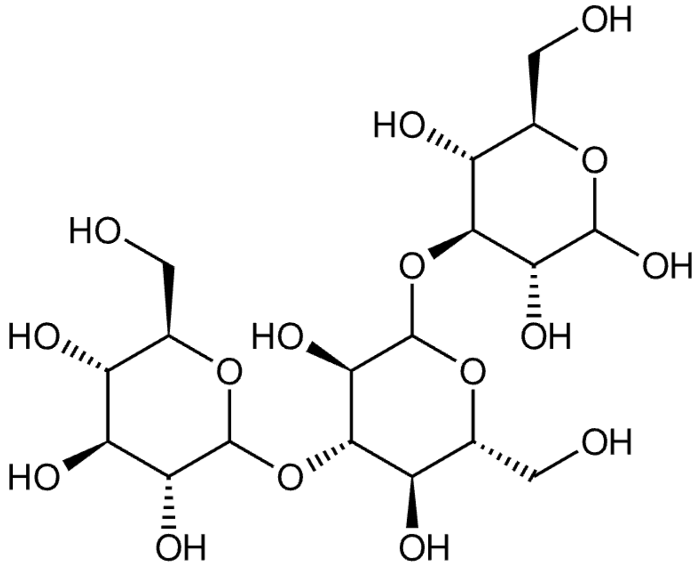
