# Supplementary material for: Exploring the antimicrobial and antibiofilm potency of four essential oils against selected human pathogens using in vitro and in silico approaches
Source: PLoS One. 2025 Apr 24;20(4):e0315663. doi: 10.1371/journal.pone.0315663 (PMC12083874; doi:10.1371/journal.pone.0315663)
Supplement: Attachment — Submitted filename: Response to Reviewer-3.pdf [file pone.0315663.s012.pdf]

Response to Reviewer

Comment Source	Comment	Response
**Reviewer**	**1. Abstract**<br>- Conciseness: While the abstract is comprehensive, consider shortening details like specific ZID ranges. Highlight only the standout findings for the antimicrobial and antibiofilm activities.<br>- Emphasis on Novelty: Stress how this study is different from prior research. For example, emphasize unique findings about the least-studied oil (CTLO) or novel in silico targets identified.	Thank you for your insightful comments and suggestions. We have revised the abstract as follows:<br>1. **Conciseness**:<br>○ Details such as specific inhibition zone diameters (ZIDs) for each pathogen have been streamlined to ensure clarity and brevity while preserving essential findings.<br>2. **Emphasis on Novelty**:<br>○ Greater focus has been placed on distinguishing this study from prior research, particularly highlighting the novel observations about citronella oil (CTLO), a relatively underexplored oil, and its unique in silico interactions.
	**2. Introduction**<br>- Rationale for EO Selection: Clearly justify why these four essential oils (CBO, BSO, CNBO, and CTLO) were selected over others. Include citations if they represent understudied oils for MDR pathogens.<br>- Highlight Problem Statement: Expand on the implications of MDR pathogens globally, particularly	Thank you for your valuable feedback on the introduction. The following revisions were made to address your concerns:<br>1. **Rationale for EO Selection**:<br>○ A dedicated section now elaborates on why the four essential oils were chosen, emphasizing their reported antimicrobial properties and highlighting the relatively understudied nature of citronella oil (CTLO). Appropriate citations were included.

Comment Source	Comment	Response
	connecting this to the inadequacy of existing treatments.<br>- Define Objective: The objective is stated, but explicitly mention how the in vitro and in silico approaches complement each other to strengthen the study's design.	**2. Problem Statement**:<br>○ The introduction expands on the global implications of MDR pathogens, with a clearer connection to the limitations of current treatments.<br>**3. Objective Definition**:<br>○ The study's objective explicitly states how the *in vitro* and *in silico* approaches complement each other, strengthening the rationale for the study design.
	**3. Materials and Methods**<br>- Reproducibility:<br>- Clarify details about EO purity for CTLO and others (e.g., confirm "stated purity ~100% (v/v)" through independent tests).<br>- Specify how EO dilutions (e.g., using DMSO) maintain stability during experiments.<br>- Target Pathogens: Provide reasoning for selecting the 19 test pathogens (e.g., their clinical relevance or prevalence in MDR infections).<br>- In Silico Docking:<br>- Expand on why these particular target proteins were chosen. For instance, describe their role in biofilm formation or MDR mechanisms.	The following revisions have been incorporated into the Materials and Methods section:<br>**1. Reproducibility**:<br>○ Purity details for all EOs were provided by the registered vendors and verified through vendor documentation and certificates of analysis.<br>○ EO dilutions were prepared in DMSO, which was included as a negative control during antimicrobial activity assays to confirm that it did not interfere with the results or affect the observed activity of the oils.<br>**2. Target Pathogens**:<br>○ A rationale for selecting the 19 test pathogens was added, emphasizing

Comment Source	Comment	Response
	- Address how docking scores were validated (e.g., benchmarking vs. known inhibitors).	their clinical relevance and association with MDR infections.<br>**3. *In Silico* Docking**:<br>○ The selection of target proteins was explained based on their role in MDR mechanisms and biofilm formation.<br>○ Docking score validation methods, including re-docking and benchmarking, were detailed.
	4. Results<br>- Presentation:<br>- Use more comparative visuals (graphs or heatmaps) for ZID, MIC, MBC, MBIC, and MBEC data instead of relying solely on tables.<br>- Highlight key results with bolded text or annotations in tables/figures, such as CNBO's superior antifungal efficacy.<br>- Insights into Antibiofilm Activity:<br>- Discuss why CTLO showed only antifungal activity but lacked antibacterial effects. Is this linked to its active components (e.g., citronellal/linalool)?<br>- Relate in vitro MBIC/MBEC findings to molecular docking results to provide mechanistic explanations.	○ As suggested, we have replaced the conventional tabular representation with comparative visuals, including heatmaps, to provide a clearer and more intuitive understanding of the ZID, MIC, MBC, MBIC, and MBEC data. These visuals allow readers to easily identify trends and compare the efficacy of various compounds.<br>○ Key results have been highlighted through annotations in these figures to emphasize their significance.<br>○ The selective antifungal activity of CTLO can be attributed to its major components, citronellal and linalool, which disrupt fungal cell membranes by targeting ergosterol and altering membrane fluidity. These components lack the specificity or potency required to disrupt bacterial membranes, which have different structural features like peptidoglycan. In contrast, CNBO's superior activity is due to its high cinnamaldehyde content, known for

Comment Source	Comment	Response
		its broad-spectrum antimicrobial properties. We have added this mechanistic explanation to the discussion section to address this observation clearly.<br>○ We have revised the discussion to link the *in vitro* MBIC/MBEC findings with molecular docking results. For example, the strong antibiofilm activity of eugenol and cinnamaldehyde correlates with their high binding affinities and stabilizing interactions with biofilm-associated proteins, while the moderate efficacy of citronellal and linalool aligns with their weaker docking interactions. These findings provide mechanistic insights into the antibiofilm potential of the essential oils, as reflected in both in vitro and in silico analyses.
	5. Discussion<br>- Contextualize Findings:<br>- Link results to the mechanism of action suggested by molecular docking (e.g., protein binding insights).<br>- Discuss CNBO's higher antifungal activity—perhaps due to cinnamaldehyde's known mechanisms.<br>- Explore why Gram-negative bacteria are generally less susceptible.<br>- Strengthen Comparison to Literature: While the discussion cites many	○ We have revised the discussion to explicitly link the *in vitro* findings to the molecular docking results, highlighting how the active ingredients (cinnamaldehyde, eugenol, thymoquinone, citronellal, and linalool) contribute to the antimicrobial and antibiofilm activities.<br>○ We have also addressed CNBO's higher antifungal efficacy by linking it to cinnamaldehyde's known ability to disrupt fungal membranes and induce oxidative stress.<br>○ The reduced susceptibility of Gram-negative bacteria has been explained

<table>
<tr><th>Comment Source</th><th>Comment</th><th>Response</th></tr>
<tr><td rowspan="3"></td><td>studies, explicitly state how your findings advance prior knowledge (e.g., new insights about the efficacy of TQ from BSO against biofilms).

- Applications: Suggest practical applications of your findings, such as potential therapeutic formulations or incorporation into healthcare settings.</td><td>by their outer membrane and LPS layer acting as barriers.
○ Lastly, we included the novelty of BSO's antibiofilm activity and practical applications, such as the potential use of EOs in antimicrobial coatings and therapeutic formulations.</td></tr>
<tr><td>6. Conclusion

- Impact Statement: Emphasize the translational potential of these EOs as alternative therapies. Mention their utility in tackling MDR biofilms specifically.

- Future Directions:

- Highlight the need for in vivo studies to confirm safety and efficacy.

- Suggest further molecular studies to understand the resistance mechanisms in pathogens where CTLO was ineffective.</td><td>○ We have revised the conclusion to address your feedback by emphasizing the translational potential of EOs, their specific utility against MDR biofilms, and the importance of future research directions.</td></tr>
<tr><td>General Inputs

- Statistical Analysis: Ensure p-values for the post-hoc analysis (Tukey's) are included alongside results to reinforce statistical rigor.

- Clarity in Figures and Tables:

- Avoid crowding tables with excessive numerical details. Use summaries with expanded legends.</td><td>○ We acknowledge and appreciate the reviewer's suggestion to include p-values for post-hoc analyses. These values are already included in the statistical analysis section to ensure clarity and statistical rigor. F-values and associated p-values for one-way ANOVA are presented for each essential oil, and post-hoc Tukey's test p-values for pairwise comparisons are explicitly provided, including instances of non-</td></tr>
</table>

Comment Source	Comment	Response
	- Provide 2D and 3D molecular docking visuals alongside in vitro results for easy cross-reference.<br><br>- Limitations: Explicitly state study limitations, such as the inability to test synergistic effects between EOs.	significant differences (e.g., BSO and CNBO for MRSA-2; p = 1.000). Detailed statistical results can be found in Tables S1 and S2 in the supplementary material. We trust this adequately addresses the reviewer's concerns.<br><br>**1. Avoid crowding tables with excessive details**:<br>○ The main tables have been summarized to present key results, while detailed numerical data are now available in the supplementary materials. Expanded legends have been included to provide clear explanations for the figures and tables.<br><br>**2. Provide 2D and 3D molecular docking visuals**:<br>○ We have incorporated key 2D and 3D molecular docking visuals into the revised figures, aligning them with the *in vitro* results for ease of cross-referencing. These visuals highlight key molecular interactions (e.g., hydrogen bonding, hydrophobic interactions) to enhance the interpretability of the docking data.<br><br>**3. Limitations:**<br><br>○ We acknowledge the importance of explicitly stating the study's limitations. The revised manuscript now includes a section discussing the inability to test synergistic effects

Comment Source	Comment	Response
		between EOs and the lack of *in vivo* validation. These limitations provide context for the scope of our findings and emphasize the need for further research in these areas.
