# Supplementary material for: Exploring the antimicrobial and antibiofilm potency of four essential oils against selected human pathogens using in vitro and in silico approaches
Source: PLoS One. 2025 Apr 24;20(4):e0315663. doi: 10.1371/journal.pone.0315663 (PMC12083874; doi:10.1371/journal.pone.0315663)
Supplement: Attachment — Submitted filename: Reviewers Correction.docx [file pone.0315663.s011.docx]

Reviewers Corrections

1. Abstract

- Conciseness: While the abstract is comprehensive, consider shortening details like specific ZID ranges. Highlight only the standout findings for the antimicrobial and antibiofilm activities.

- Emphasis on Novelty: Stress how this study is different from prior research. For example, emphasize unique findings about the least-studied oil (CTLO) or novel in silico targets identified.

2. Introduction

- Rationale for EO Selection: Clearly justify why these four essential oils (CBO, BSO, CNBO, and CTLO) were selected over others. Include citations if they represent understudied oils for MDR pathogens.

- Highlight Problem Statement: Expand on the implications of MDR pathogens globally, particularly connecting this to the inadequacy of existing treatments.

- Define Objective: The objective is stated, but explicitly mention how the in vitro and in silico approaches complement each other to strengthen the study’s design.

3. Materials and Methods

- Reproducibility:

- Clarify details about EO purity for CTLO and others (e.g., confirm "stated purity ~100% (v/v)" through independent tests).

- Specify how EO dilutions (e.g., using DMSO) maintain stability during experiments.

- Target Pathogens: Provide reasoning for selecting the 19 test pathogens (e.g., their clinical relevance or prevalence in MDR infections).

- In Silico Docking:

- Expand on why these particular target proteins were chosen. For instance, describe their role in biofilm formation or MDR mechanisms.

- Address how docking scores were validated (e.g., benchmarking vs. known inhibitors).

4. Results

- Presentation:

- Use more comparative visuals (graphs or heatmaps) for ZID, MIC, MBC, MBIC, and MBEC data instead of relying solely on tables.

- Highlight key results with bolded text or annotations in tables/figures, such as CNBO’s superior antifungal efficacy.

- Insights into Antibiofilm Activity:

- Discuss why CTLO showed only antifungal activity but lacked antibacterial effects. Is this linked to its active components (e.g., citronellal/linalool)?

- Relate in vitro MBIC/MBEC findings to molecular docking results to provide mechanistic explanations.

5. Discussion

- Contextualize Findings:

- Link results to the mechanism of action suggested by molecular docking (e.g., protein binding insights).

- Discuss CNBO’s higher antifungal activity—perhaps due to cinnamaldehyde’s known mechanisms.

- Explore why Gram-negative bacteria are generally less susceptible.

- Strengthen Comparison to Literature: While the discussion cites many studies, explicitly state how your findings advance prior knowledge (e.g., new insights about the efficacy of TQ from BSO against biofilms).

- Applications: Suggest practical applications of your findings, such as potential therapeutic formulations or incorporation into healthcare settings.

6. Conclusion

- Impact Statement: Emphasize the translational potential of these EOs as alternative therapies. Mention their utility in tackling MDR biofilms specifically.

- Future Directions:

- Highlight the need for in vivo studies to confirm safety and efficacy.

- Suggest further molecular studies to understand the resistance mechanisms in pathogens where CTLO was ineffective.

General Inputs

- Statistical Analysis: Ensure p-values for the post-hoc analysis (Tukey's) are included alongside results to reinforce statistical rigor.

- Clarity in Figures and Tables:

- Avoid crowding tables with excessive numerical details. Use summaries with expanded legends.

- Provide 2D and 3D molecular docking visuals alongside in vitro results for easy cross-reference.

- Limitations: Explicitly state study limitations, such as the inability to test synergistic effects between EOs.
